# Nutritional control of gene expression in *Drosophila *larvae via TOR, Myc and a novel cis-regulatory element

**DOI:** 10.1186/1471-2121-11-7

**Published:** 2010-01-20

**Authors:** Ling Li, Bruce A Edgar, Savraj S Grewal

**Affiliations:** 1 Fred Hutchinson Cancer Research Center, Seattle, WA 98109, U.S.A; 2 Clark H. Smith Brain Tumour Center, Southern Alberta Cancer Research Institute, Calgary, Alberta, T2N 4N1, Canada; 3 Department of Biochemistry and Molecular Biology, University of Calgary, Calgary, Alberta, T2N 4N1, Canada

## Abstract

**Background:**

Nutrient availability is a key determinant of eukaryotic cell growth. In unicellular organisms many signaling and transcriptional networks link nutrient availability to the expression of metabolic genes required for growth. However, less is known about the corresponding mechanisms that operate in metazoans. We used gene expression profiling to explore this issue in developing *Drosophila *larvae.

**Results:**

We found that starvation for dietary amino acids (AA's) leads to dynamic changes in transcript levels of many metabolic genes. The conserved insulin/PI3K and TOR signaling pathways mediate nutrition-dependent growth in *Drosophila *and other animals. We found that many AA starvation-responsive transcripts were also altered in TOR mutants. In contrast, although PI3K overexpression induced robust changes in the expression of many metabolic genes, these changes showed limited overlap with the AA starvation expression profile. We did however identify a strong overlap between genes regulated by the transcription factor, Myc, and AA starvation-responsive genes, particularly those involved in ribosome biogenesis, protein synthesis and mitochondrial function. The consensus Myc DNA binding site is enriched in promoters of these AA starvation genes, and we found that Myc overexpression could bypass dietary AA to induce expression of these genes. We also identified another sequence motif (Motif 1) enriched in the promoters of AA starvation-responsive genes. We showed that Motif 1 was both necessary and sufficient to mediate transcriptional responses to dietary AA in larvae.

**Conclusions:**

Our data suggest that many of the transcriptional effects of amino acids are mediated via signaling through the TOR pathway in *Drosophila *larvae. We also find that these transcriptional effects are mediated through at least two mechanisms: via the transcription factor Myc, and via the Motif 1 cis-regulatory element. These studies begin to elucidate a nutrient-responsive signaling network that controls metabolic gene transcription in *Drosophila*.

## Background

The availability of extracellular nutrients is a key determinant of eukaryotic cell growth. In single cell organisms, such as budding yeast, an extensive signal transduction and transcriptional network links extracellular nutrients to the expression of metabolic genes [1,2]. These networks are essential for the proper control of cell growth and proliferation [3,4]. In metazoans, nutrition controls both cell and organismal growth. These effects require a complex interplay between cell-autonomous and systemic responses to nutrient availability. However, the cellular mechanisms that mediate these effects are still poorly understood.

In *Drosophila *dietary amino acids are essential for larval growth and development [5]. Amino acid starvation leads to inhibition of cell growth and cell cycle progression in virtually all larval tissues. The *Drosophila *insulin/PI3 kinase pathway plays a central role in nutrition-regulated growth [6]. In response to dietary protein, *Drosophila *insulin-like peptides (Dilps) are released from neurosecretory cells. These Dilps act in an endocrine manner and trigger growth by binding to the insulin receptor and activating a conserved PI3 kinase (PI3K) and Akt kinase signaling pathway in all larval tissues [7]. The TORC1 (TOR complex 1) pathway is a second important mediator of nutritional inputs. The TORC1 protein complex contains the TOR kinase and can be activated in a cell autonomous manner in response to extracellular nutrients and amino acids, and also as a downstream target of the insulin/PI3K pathway [8,9]. As such, insulin/PI3K and TOR can function either independently or as a linear signaling cascade, depending on the stimulatory inputs. Importantly, cell autonomous activation of both insulin/PI3K and TOR can bypass the requirement for dietary protein to control growth in *Drosophila *larval tissues [6,10,11].

Much is known about the complex signaling inputs and crosstalk between the insulin/PI3K and TOR pathways. These regulatory inputs are highly conserved and control cell and organismal growth in all animals. Many oncogenes and tumor suppressors are part of these signaling inputs, and aberrant PI3K and TOR signaling is a feature of many different cancers [12]. However, less is known about the growth regulatory outputs of these pathways [13]. Given the important role of transcription in mediating effects of nutrients in budding yeast it is likely that changes in gene expression are important in metazoans too. Recent studies in *Drosophila *support this notion [13-17]. For example, the FOXO transcription factor, a conserved target of the insulin/PI3K pathway, can regulate gene expression in response to starvation in larvae, in part through secondary regulation of the transcription factor Myc [16]. Also, nutrient-dependent TOR signaling controls ribosomal RNA (rRNA) transcription in larvae, via the conserved Pol I factor, TIF-IA [18]. Finally, in *Drosophila *adults, brain specific insulin/PI3K signaling can control gene expression through the transducer of regulated CREB (TORC) transcriptional regulator [19].

In this paper we report gene expression profiling of both AA starvation, and the PI3K and TOR pathways in *Drosophila *larvae. We find that starvation for dietary AA induces robust and dynamic changes in metabolic gene expression. By comparing gene expression profiles we suggest that some, but not all, transcriptional effects of starvation are mediated through the TOR pathway. Interestingly, although overexpression of PI3K induces robust changes in metabolic gene expression, these changes show a limited overlap with the AA starvation expression profile. Instead, we find that many of the transcriptional responses to changes in dietary protein are mediated via the transcription factor dMyc. Finally, we identified DNA motifs in promoters of protein-responsive genes and show that a previously uncharacterized motif (Motif 1) confers AA starvation-responsiveness in transgenic reporter lines. Together these studies begin to elucidate a nutrient-responsive signaling network that controls metabolic gene transcription in *Drosophila*.

## Results

### Gene expression profiling in protein-starved larvae

Starvation for dietary amino acids (AAs) leads to growth arrest and cell cycle inhibition in virtually all polyploid cells of the larval organs [5]. We were therefore interested in identifying AA starvation-induced transcriptional changes occurring in the bulk of these tissues. Thus we used cDNA microarrays to analyze gene expression profiles in whole larvae. We starved second instar larvae by floating them in 20%sucrose/PBS at 62 h +/- 3 hrs after egg deposition, and then examined gene expression profiles at various time points following AA starvation. For all time points, comparisons were made to zero hour time point control larvae.

**Figure 1:**
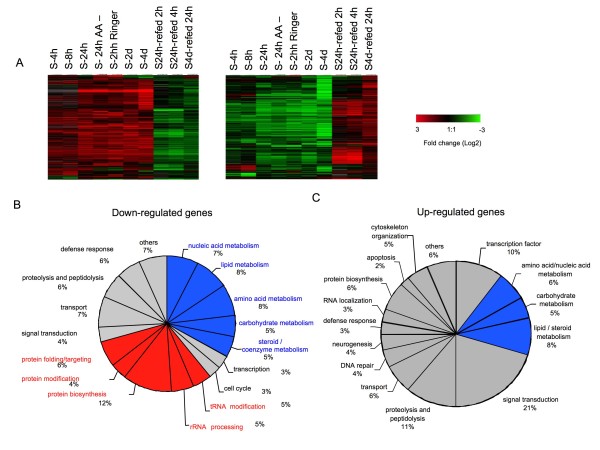
**Protein starvation regulates gene expression in Drosophila larvae**. A. Heat map depicting temporal patterns of changing gene expression following starvation and subsequent re-feeding. Left panel, transcripts induced by starvation and reversed by re-feeding. Right panel, transcripts suppressed by starvation and reversed by re-feeding. Columns indicate expression changes at different AA starvation/re-feeding time-points B. Gene classes whose expression was down-regulated at 24 h following protein starvation. Blue shading indicates metabolic genes, red shading indicates ribosome and/or protein synthesis genes. C, Gene classes whose expression was up-regulated at 24 h following protein starvation. Blue shading indicates metabolic genes.

Of the six thousand genes from DGC-1 on the microarray chips, Significance Analysis of Microarrays (SAM) analysis indicated that a total of 1888 were altered by AA starvation (Table 1 and Additional File 1). We observed both increases and decreases in gene expression. Significant changes were seen at four-hour starvation suggesting a very rapid transcriptional response to AA starvation. Strong effects were seen at 4 days post starvation with decreases in 636 transcripts and increases in 647 transcripts, which together make up almost a quarter of all genes analyzed on the microarrays (Figure 1 and Table 1). Importantly, most AA-starvation changes in transcript levels were reversed upon re-feeding (Figure 1A). For example, half (172 of 325, 53%) the genes that were either suppressed or enhanced at 24 h of AA starvation were reversed within 4 h of re-feeding larvae with a protein rich diet. Similarly, one-day of re-feeding reversed many (254 of 636, 40%) changes in gene expression caused by 4-day starvation. Cluster analysis identified several groups of genes with different expression kinetics. These clusters suggest a dynamic temporal pattern of gene expression in response to AA starvation and re-feeding (Additional File 2).

**Table 1:** Summary of AA starvation-regulated genes.

	Down-regulated genes	Up-regulated genes	Total	% of total genes on the microarray
AA starved 4 h	210	313	523	9.4
AA starved 8 h	203	226	429	7.7
AA starved 24 h	325	260	585	10.6
Complete food minus AA 24 h	256	267	523	9.4
Complete starve 24 h	350	358	708	12.8
AA starved 2 d	434	393	827	14.9
AA starved 4 d	636	647	1283	23.2
AA Starved 24 h Refed 2 h	138	274	412	7.4
AA Starved 24 h Refed 4 h	202	378	580	10.4
AA Starved 4 d Refed 1 d	115	313	428	7.7

Based on the types of genes affected, our data pointed to broad transcription-dependent changes in cellular and organismal metabolism upon AA starvation. For example, at 24 h starvation 325 transcripts were reduced. Of these 156 were of unknown function (i.e. no Gene Ontology (GO) annotation). However, of the transcripts with GO term annotations, approximately one-third were involved in ribosome biogenesis and protein synthesis while another one-third of reduced transcripts were annotated as being involved in nutrient metabolism, including many genes required for mitochondrial function (Figure B and Additional File 3). Many genes involved in proteolysis were also altered in response to amino acid withdrawal (Additional File 4). In particular, several autophagy related genes (ATGs) were induced, in some cases within 4-8 h of protein starvation (Additional File 4). Expression of genes coding for regulators of lipid and steroid metabolism was also affected by starvation (Additional File 5). Interestingly although AA starvation leads to a block in cell cycle progression in most larval tissues, particularly the endoreplicating tissues which make up the bulk of the animal, transcripts coding for cell cycle genes were largely unaffected by starvation (Additional File 6). Together these data suggest a dynamic, rapid transcriptional response to protein starvation that both increases and decreases gene expression and alters larval metabolism.

We also compared the gene expression profiles obtained from three different 24 hr starvation conditions (complete starvation, sucrose-only diet, standard diet minus protein) (Additional File 7). In general there was a strong overlap between affected genes across each condition, particularly with respect to genes whose expression was down-regulated. For example, 65% of transcripts whose expression was reduced upon complete starvation were also suppressed in the sucrose-only condition. These data suggest that a key, limiting factor for gene expression is availability of dietary AA (Figure 1A and Additional File 7). Nevertheless, expression of many genes was affected in only one of the starvation conditions, possibly reflecting an additional role for dietary sugars and fats in the control of gene expression. This was particularly true for transcripts whose expression was increased upon starvation (Additional File 7).

### Transcriptional changes induced by PI3K and TOR signaling pathways

**Figure 2:**
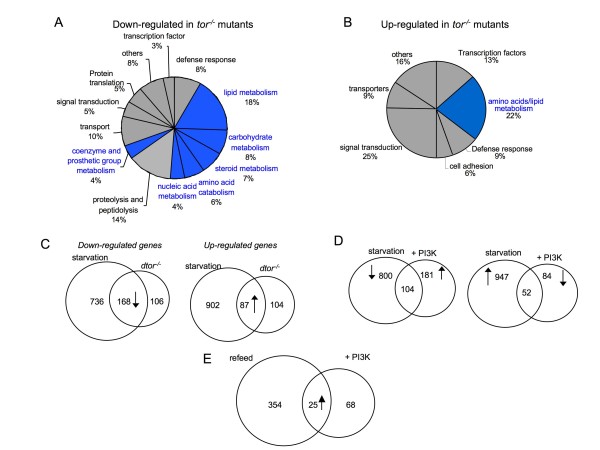
**Expression profiling of TOR mutants and PI3 kinase overexpression**. A. Gene classes whose expression was down-regulated in *tor*^-/- ^mutants. Blue shading indicates metabolic genes. B, Gene classes whose expression was upregulated in *tor*^-/- ^mutants. Blue shading indicates metabolic genes. C, Venn diagram showing the overlap in genes regulated by protein starvation and TOR mutants. Left panel, genes down-regulated in both conditions. Right panel, genes up-regulated in both conditions. D, Venn diagram showing the overlap in genes whose expression is reciprocally regulated by protein starvation and PI3 kinase overexpression. E. Venn diagram showing the overlap in genes regulated in AA-starved animals by either re-feeding with complete food or by PI3 kinase overexpression.

We next examined the gene expression profiles associated with the TOR and insulin/PI3K signaling pathways. A null allele of TOR phenocopies AA-starvation and leads to growth arrest in early larval development (~72 h after egg deposition (AED) [20]. Expression profiling revealed that a total of 464 transcripts were significantly altered in *tor*^-/- ^mutant larvae at 72 h AED (Figure 2A, B). As with AA starvation, these transcripts coded for genes largely involved in metabolism (Figure 2A, B and Additional File 1), although considerably fewer transcripts were altered in *tor*^-/- ^mutants. Nevertheless, 61% of transcripts that were significantly reduced in *tor*^-/- ^mutants were also significantly reduced in protein-starved larvae (Figure 2C). These altered transcripts comprised approximately 19% of starvation-suppressed transcripts. In addition 46% of transcripts increased in *tor*^-/- ^mutants were also increased in starved larvae. This suggests that inhibition of TOR partially mediates the transcriptional response to AA starvation.

The Insulin/PI3K pathway is activated in response to nutrient availability, and is thought to be an important regulatory input to the TOR pathway. We over expressed a PI3K transgene in third instar larvae and examined gene expression profiles at 14 and 24 h post-induction. At the early time, expression levels of 118 transcripts were altered (87 increased and 39 decreased), whereas at 24 h this increased to 421 transcripts (285 increased and 341 decreased). The bulk of the altered transcripts coded for genes required for carbohydrate and lipid metabolism (Additional File 1 and Additional File 8). Other affected genes were involved in protein biosynthesis, transcription and mitochondrial function (Additional File 1 and Additional File 8).

Given the role of PI3K in nutrition-dependent signaling, we predicted a reciprocal relationship between genes affected by PI3K overexpression and starvation. However, of the 285 genes increased at 14 h post PI3K-induction in fed animals, only 48 (17%) were decreased in 24 h AA-starved larvae (48% of all 24 h starvation suppressed transcripts) (Figure 2D). We also compared gene expression profiles of 24 h AA starved larvae that were either refed or in which PI3K was overexpressed for 14 hrs. As described previously, 4 hr of re-feeding reversed 52% of the 24 h starvation-mediated changes in gene expression. However, we found only a small overlap (17%) in the re-feeding expression profiles versus the expression profile associated with overexpressing PI3K in starved animals (Figure 2E). These data suggest that only a small fraction of AA starvation-regulated genes respond to overactivation of the insulin/PI3K pathway.

### Myc regulates the expression of starvation responsive genes

We previously showed that overexpression of the transcription factor Myc in larvae could increase the expression of many metabolic genes, particularly those involved in ribosomal biogenesis and protein synthesis genes [21]. These Myc regulated genes strongly overlapped with many AA starvation-responsive genes identified in this report. For example, 60% of genes induced by AA-starvation and re-feeding are also induced by 14 hr Myc overexpression in larvae. This overlap in regulation was particularly true for genes involved in ribosome biogenesis and protein synthesis (Figure 4). A MEME motif search identified the consensus Myc binding site (E-box - CACGTG) as being highly enriched in promoters of Pol I and Pol III genes and rRNA processing genes, and genes required for mRNA translation. Interestingly, although the ribosomal protein genes were all regulated by Myc, very few had canonical Myc E-boxes in their promoter. This suggests Myc may function via intermediate transcription factors to control the expression of ribosome protein genes.

**Figure 3:**
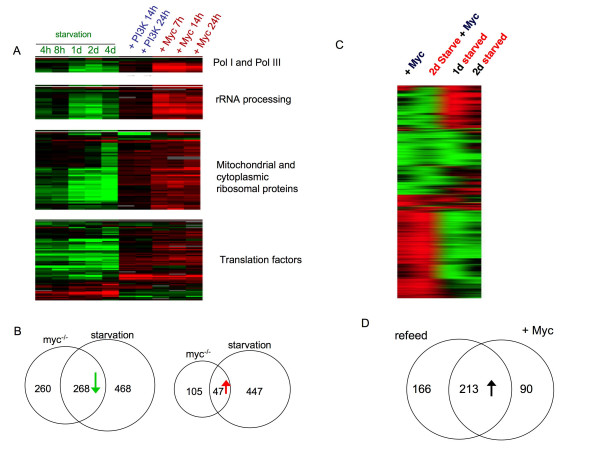
**Myc and protein starvation regulate similar genes, particularly those involved in ribosome synthesis and mRNA translation**. A. Heat map of ribosome biogenesis and mRNA translation genes. Each column represents change in gene expression following starvation, Myc overexpression or PI3K overexpression. Columns indicate expression changes at different time-points B, Venn diagram showing overlap in genes whose expression is either decreased (left panel) or increased (right panel) in both Myc null mutants and protein starved mutants. C. Heat map of genes affected by Myc overexpression, starvation or Myc overexpression in starved larvae. D. Venn diagram showing the overlap in genes up-regulated in starved animals by either re-feeding or by Myc overexpression.

We also compared the gene expression profiles of Myc homozygous null mutant and AA-starved animals. Myc null mutants are lethal and arrest their growth early in larval development, and show changes in gene expression that are largely the reciprocal of those mediated by Myc overexpression [22]. We identified a strong overlap between genes whose transcripts levels were reduced in both AA-starved animals and 24 h *myc*^-/- ^homozygous mutant larvae (51% of Myc-regulated genes) (Figure 3B). Of the genes that overlap, nearly all contain E-boxes (including half with consensus Myc binding sites -CACGTG). This overlap suggests that Myc may mediate transcriptional activation induced by AA-dependent signaling. In contrast less then 10% of genes whose expression was increased upon AA-starvation were altered in *myc*^-/- ^mutants.

**Figure 4:**
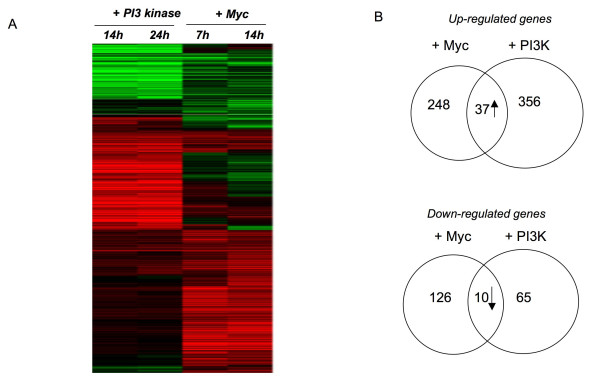
**Myc and PI3 kinase overexpression regulate different genes**. A, Heat map of genes affected by either Myc or PI3 kinase overexpression. Columns indicate expression changes at different time-points following either Myc or PI3 kinase expression. B, Venn diagram showing overlap in genes whose expression is either increased (upper panel) or decreased (lower panel) following either Myc or PI3 kinase overexpression in larvae.

To further examine the role of Myc in AA-dependent transcription we analyzed the gene expression profiles in 24 h AA starved larvae in which we over expressed a Myc transgene for a further 24 h. We found that many of the changes in gene expression induced by starvation could be reversed by overexpression of Myc (Figure 3C). In particular, we found that 70% of transcripts increased by Myc overexpression in AA-starved larvae were also increased upon re-feeding in starved animals (these genes accounted for 56% of all refeeding-induced transcripts - Figure 3D). Together, these data suggest that Myc overexpression can bypass starvation to control the expression of many AA-responsive genes. This contrasts with our findings with PI3K overexpression. Not surprisingly, we found very little overlap (9% of Myc-induced genes and 13% of Myc-repressed genes) between the gene expression profiles of Myc-versus PI3K-overexpression in third instar larvae (Figure 4)

### Other potential nutrient-dependent transcriptional regulators

**Figure 5:**
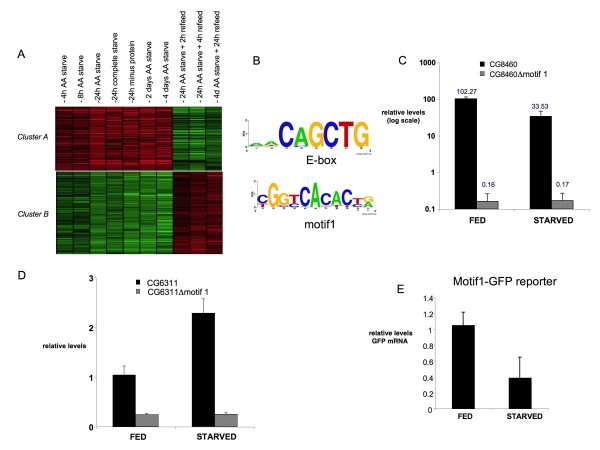
**Motif 1 is a mediator of nutrition-dependent transcription**. A. Heat-maps showing expression profiles of Cluster A and Cluster B genes. Columns indicate expression changes at different AA starvation/re-feeding time-points B. Weblogos of two motifs enriched in the promoters of AA starvation-regulated genes. C, D. Real-time qPCR data from transgenic larvae carrying promoter-GFP reporters for two different starvation-regulated genes. Black bars, wild-type promoter, Grey bars, motif1-mutated promoters. E. Real-time qPCR data from transgenic larvae carrying 5× motif1-GFP reporter.

A goal of these studies was to identify potential cis-regulatory DNA motifs and corresponding transcription factors that control transcription of nutrient-regulated genes. As described above Myc is a strong candidate and many AA starvation-regulated genes contained canonical Myc E-boxes within their promoters. We used the MEME motif search tool to try and identify other motifs enriched within the promoters of AA starvation-responsive genes. We analyzed two clusters of genes whose expression profiles showed strong regulation by AA starvation (Figure 5A). Cluster A contained genes whose transcript levels were rapidly induced and maintained by AA starvation, and then rapidly decreased by AA refeeding (Fig 5A). Cluster B contained genes whose transcripts were regulated in the opposite manner. Two of the top motifs identified in this bioinformatic search were an E-box bHLH transcription factor binding site (found in 14/42 Cluster A genes and 27/46 of Cluster B genes, p < 5 × 10^-4^), and a previously identified motif, Motif 1, (23/42 Cluster A genes and 21/46 of Cluster B genes p < 6.4 × 10^-6^) (Figure 5B). We found that 9/46 cluster B genes and 10/46 cluster A genes contained both motifs. We also performed a similar MEME analysis on four non-overlapping groups of 40 genes whose expression was not altered by AA starvation (data not shown). In each case MEME analysis did not identify either Motif1 or an E-box in the promoters of these genes, suggesting that both motifs may be enriched in promoters of starvation-regulated genes. Motif 1 was previously identified as a core promoter element of many *Drosophila *genes [23] and we found it was close to the transcription start site of both Cluster A and B genes. In order to test the importance of Motif 1, we generated promoter-GFP reporter lines from seven genes from both cluster A and B (cluster A - CG7686, CG1135, CG6311, CG32486; cluster B - CG8470, CG6459, CG9539). In each case GFP was expressed in all tissues examined and mutation of Motif1 in each reporter abolished visible GFP expression (data not shown). We next used qRT-PCR to examine expression of GFP reporter for a gene from each of the two clusters: CG8470 from cluster B, and CG6311 from cluster A. Upon AA starvation, CG8470-GFP mRNA expression was reduced, while expression from CG6311-GFP was induced (Figure 5C, D). These changes are similar to alterations in endogenous transcript levels as measured by Northern blot (data not shown) and microarray hybridization, consistent with the notion that the AA starvation-induced changes in mRNA levels are due to alterations in transcription. When we mutated Motif 1 in both reporters, expression was markedly reduced (Figure 5C, D). More importantly, we also found that the weak GFP expression from these Motif 1 mutated reporters was no longer starvation-responsive. We next generated several transgenic lines containing reiterated Motif 1 with a basal hsp promoter. This artificial Motif1 promoter was sufficient to drive GFP expression in all larval tissues examined (wing discs, gut, fat body, salivary gland). Interestingly, we found that GFP mRNA expression from this reporter was reduced when larvae were AA-starved (Figure 5E). These data suggest Motif 1 may bind a factor(s) required for transcription of AA-starvation responsive genes and whose activity may be regulated by AA availability. We used GOMO (Gene Ontology for Motifs) analysis to identify GO-terms that are significantly associated with genes containing motif 1 within their upstream regions (Additional File 9). The top scoring hits in this analysis were genes that were broadly involved in cell metabolism and cell division, based on GO definitions of molecular function and biological process. Interestingly, GOMO analysis also identified genes involved in ribosome synthesis based on the GO term of cellular component (Additional File 9). 3). We examined this further using MEME analysis of the upstream regions of ribosome biogenesis gene. Of the cluster of 82 ribosome synthesis genes that we examined, we identified both motif 1 (44 genes, p < 1 × 10^-27^) and the consensus Myc E-box (42 genes, p < 5 × 10^-9^) as two of the top motifs, with 13 genes containing both motifs. Together these analyses suggest that transcription through an E-box and/or motif1 links AA-dependent signaling to metabolic gene expression, particularly genes involved in ribosome biogenesis and protein synthesis.

## Discussion

### Nutritional control of gene expression in Drosophila larvae

Starvation for dietary AA leads to profound changes in metabolism of *Drosophila *larvae. These changes include decreases in ribosome and protein synthesis [18,24,25], alterations in the storage and metabolism of fat and carbohydrate [6,25,26], and induction of autophagy [27]. These alterations are essential for arresting larval growth and development, and maintaining homeostasis under poor nutrient conditions[24,25,28]. Our microarray data suggest that many of these changes are mediated through nutrient-dependent modulation of gene expression. For example, mRNA levels of both protein synthesis and ribosome biogenesis genes are reduced upon AA starvation. Similarly genes involved in mitochondrial biogenesis and function, such as mitochondrial ribosomal proteins and a mitochondrial transcription factor, TFAM, are also suppressed by AA starvation. In contrast, several genes involved in proteolysis and autophagy are rapidly induced by removal of dietary AA.

A previous report also described both protein and complete nutrient starvation responses in whole larvae [14]. Although this study used different protocols to analyze gene expression, similar, but fewer, genes were identified compared to our study. More recently, Telemann et al (2008) [16] examined transcriptional responses to complete starvation in either fat or muscle. They identified many genes whose regulation was specific to either tissue, but they also reported changes in similar classes of genes especially those involved in ribosome synthesis, mRNA translation and mitochondrial biogenesis. Our analyses were done using whole larvae therefore we are probably identifying strong transcriptional changes that are occurring in most larval cells. Together, these previous reports and our data identify the major changes in metabolic gene expression mediated by dietary AA in *Drosophila *larvae.

The bulk of the larval organs (such as the gut, fat body and muscle) are composed of polyploid cells which all undergo nutrition-dependent endoreplication [5,29]. AA starvation leads to a shutdown of cell cycle progression in these tissues [5,29]. However, our microarray analyses on starved larvae did not reveal significant changes in mRNA levels of cell cycle regulators. This finding suggests that nutrition-dependent control of cell cycle progression may occur through post-transcriptional changes in cell cycle genes. This type of regulation has been described in yeast in which nutrient-dependent alterations in ribosome synthesis and mRNA translation can control translation of cell cycle genes [30].

### Transcript profiling of the PI3K and TOR signaling pathways

The insulin/PI3K and TOR signaling pathways are the major mediators of nutrition-dependent cell and organismal growth in *Drosophila *larvae. Our microarray analyses allowed us to compare the expression profiles of AA starvation with modulation of PI3K and TOR signaling. These comparisons suggest that only about half of genes whose expression is altered by AA starvation also respond to modulation of PI3K and/or TOR signaling. The strongest overlap was between TOR and AA starvation-regulated genes. Virtually all of these overlapping genes were involved in the control of some aspect of cellular metabolism, particularly the control of carbohydrate and lipid metabolism. However, we did see some notable differences between starvation and TOR mutants. In particular, TOR mutants showed little change in the expression of ribosome biogenesis and protein synthesis genes. TOR mutants have reduced nucleolar size and lower levels of rRNA synthesis [18,20], two effects that are indicative of reduced ribosome synthesis and that are phencopied by protein starvation [18]. Also, inhibition of TOR in cultured *Drosophila *cells does inhibit expression of ribosome biogenesis genes [31]. It is possible these differences may reflect tissue specific effects (i.e. cell culture versus larvae). However, it is likely that many of the effects of TOR on ribosome synthesis may be mediated through post-transcriptional control of downstream effectors e.g. TIF-IA [18] and 4E-BP. Indeed many of the nutrition-dependent functions of TOR probably reflect transcription-independent effects of TOR on processes such as mRNA translation [32], autophagy [28] and endocytosis [33].

We saw a much weaker overlap between genes affected by AA starvation versus PI3K overexpression. For example, given the role of insulin/PI3K signaling in nutrition-dependent growth, we predicted that genes suppressed by starvation might be induced by PI3K overexpression. However, this prediction was correct for only a small subset (12%) of AA-starvation suppressed genes. In particular we found that genes involved in ribosome biogenesis and protein synthesis, two processes strongly influenced by protein starvation, were generally unaffected by PI3K. Thus, not all AA starvation-regulated genes respond to activation of the insulin/PI3K pathway. The transcription factor FOXO is a conserved downstream target of the PI3K pathway. High PI3K activity leads to nuclear export and inhibition of FOXO [34]. It has been suggested that many transcriptional responses to PI3K activation are mediated through repression of FOXO activity. Indeed, a recent paper implicated FOXO in mediating many transcriptional effects of AA starvation [16]. This suggestion would seem at odds with our comparison of AA starvation and PI3K-responsive genes. These differences between our findings with PI3K overexpression and previous examination of FOXO mutants may be due to different experimental approaches. For example, we examined gene expression following overexpression of PI3K in both fed and starved larvae, whereas Telemann et al. examined starvation expression profiles in either fat or muscle of FOXO mutants. In addition, it is likely that many of the effects of PI3K overexpression on both transcription and growth can occur independently of FOXO. For example, FOXO mutants have no growth phenotypes in nutrient rich food [35], while overexpression of PI3K can induce marked cell growth under identical conditions [6], suggesting that the effects of PI3K involve more than FOXO inhibition alone.

### Myc as a downstream effector of nutrition-dependent transcription

One key finding from our work was the role of Myc in nutrition-mediated gene expression. We found that the gene expression profiles of both Myc mutants and Myc-overexpression exhibit a strong overlap with AA starvation-responsive genes. Many of the overlapping genes were involved in ribosome synthesis, mRNA translation and mitochondrial function. Moreover, we showed that Myc overexpression could bypass a requirement for AA and reverse repression of many of genes induced by starvation. These findings suggest that Myc acts as a downstream mediator of AA-dependent regulation of gene transcription and expression of metabolic genes in *Drosophila *larvae. Most of these Myc- and AA starvation-sensitive genes contain either the consensus Myc binding site or alternate E-box motifs that may also bind Myc, within their promoters. A recent paper reported a similar enrichment of Myc binding sites within the promoters of genes inhibited by rapamycin treatment in cultured *Drosophila *S2 cells, particularly ribosome synthesis genes [16]. This latter study reported that Myc association with the promoters of these genes was inhibited by treatment with the TOR inhibitor, rapamycin [16]. Our in vivo 'by-pass' experiments extend these cell culture findings to show that Myc functions downstream of nutrition to control metabolic gene expression in developing larvae. Two recent reports indicated that Myc mRNA levels were FOXO-regulated in a tissue specific manner [16,36], hence providing one potential mechanism by which Myc might be regulated downstream of AA availability. Regulation of Myc protein levels by either translation control or ubiquitin mediated-degradation may also be a downstream effect of AA.

An important finding from our work was the marked differences in the expression profiles of PI3K-overexpression versus Myc-overexpression. For example, we found that Myc induced expression of a large number ribosome biogenesis and protein synthesis genes (Fig 4) whereas PI3K-overexpression did not. Consistent with this, we previously reported that Myc overexpression had strong effects on rRNA synthesis, whereas PI3K overexpression had little or no effect [21]. Thus, while both Myc and PI3K can induce quantitatively similar increases in growth, comparisons between their gene expression profiles suggest qualitatively different effects of Myc versus PI3K on transcription and metabolism. Indeed, Myc and PI3K produce morphologically different effects on cell growth in the larval fat body [37]. Importantly, these differences between PI3K and Myc suggest that the effects of insulin/PI3K on transcription are probably in large part independent of Myc function. In contrast, a recent paper reported that in cultured *Drosophila *S2 cells, insulin treatment (and presumably increased PI3K activity) promoted Myc binding to the promoters of ribosome biogenesis genes and increased expression of these genes [16]. These results contrast with our data and may reflect differences between responses to increased PI3K activity in *in vitro *cultured S2 cells versus developing larvae. Nevertheless, our data do point to differences in the way PI3K and Myc regulate gene expression and cell growth in vivo. These qualitative differences between how PI3K and Myc function may have implications for situations in which growth is deregulated, such as cancer. Thus, increases in PI3K pathway activity and Myc may each induce distinct effects on metabolism, and consequently cooperate to drive aberrant cell growth and induce transformation [38]. In fact, increased Myc levels and overactivation of effectors of the PI3K pathway are observed in many cancers and may function synergistically to promote tumor progression [39].

### Motif1 as a AA starvation-regulated promoter element

Nutrient dependent changes in gene transcription have been well studied in budding yeast [1,2]. In this unicellular organism alterations in extracellular levels of nutrients and amino acids can elicit widespread changes in transcription of large classes of metabolic genes, particularly those involved in ribosome biogenesis and protein synthesis [2,4]. In many cases the cis-regulatory promoter elements and corresponding DNA-binding factors that mediate these responses have been defined [2]. However, these factors often have no obvious homologs in metazoans. In *Drosophila*, our work and that of others suggests Myc and TORC2-CREB function as nutrition/PI3K/TOR-regulated transcription factors [16,19]. However, no additional factors have been described. In this paper we surveyed the promoters of genes whose expression is AA starvation-sensitive for enriched DNA motifs. One motif in particular (Motif1) emerged from this analysis. Motif 1 was previously identified as a core promoter element in a large number of genes in *Drosophila*, although the exact binding factor(s) are not known [23]. Our data suggest that Motif1 is required for transcription of AA starvation-responsive genes. Moreover, Motif-dependent transcription is inhibited by AA starvation. These findings raise the possibility that motif 1 is a binding site for AA starvation-regulated transcription factor(s). It is likely that this putative factor(s) is an activator rather than a repressor since mutation of motif1 in the transgenic reporters led to reduced transcription as opposed to de-repression (and increased transcription). Given the location of motif 1 within the core promoters of many *Drosophila *genes and its proximity to the transcription start site [23], the factor(s) that bind Motif 1 may also either be, or associate with, core promoter factors. This view is consistent with emerging data suggesting that regulation of core promoter factors can control differential gene expression. For example, in mammalian cells, induction of myogenesis involves differential expression and recruitment of core transcription factors [40]. Also, in yeast the expression of stress- versus growth-regulatory genes in response to environmental cues depends on differential recruitment of core promoter factors [1]. We found Motif1 in the promoters of many classes of AA starvation-responsive metabolic genes, particularly those involved in protein synthesis and ribosome biogenesis. Moreover, a previous genome-wide binding analysis of dMyc showed that the presence of Motif1 significantly correlates with the presence of consensus Myc-binding sites [41]. These findings together with our data suggest that a putative motif-1-binding factor and Myc may function to control metabolic gene transcription in response to nutrient availability in *Drosophila*. Whether transcription through both promoter elements is cooperative or additive is unclear. However, since many AA starvation-responsive genes (e.g. cluster A and B genes, ribosome biogenesis genes) contain only one or other of the two motifs, motif1 and the E-box can probably function independently of each other.

## Conclusions

Our data show that AA starvation leads to widespread changes in metabolic gene expression in Drosophila larvae. These effects are mediated in large part through the TOR signaling pathway. We also identify at least two transcriptional mechanisms by which these changes occur: via the transcription factor, Myc, and through the Motif-1 cis-regulatory element.

## Methods

### Fly strains and protocols

The following fly stocks were used: ywhsflp122; +; +, ywhsflp122; +; UAS-dMyc, ywhsflp122; UAS-dp110, Act>CD2>GAL4, UAS-GFP, w1118; dTOR^ΔP^/CyO-GFP.

All flies were raised at 25C. For all starvation experiments wild-type embryos were collected on agar/grape juice plates. Fifty newly hatched larvae were transferred to vial of normal fly food supplemented with yeast paste. They were raised for 36 hr (+/-3 hrs) and then starved by first washing with 1× PBS and then floating in 20% sucrose/PBS. Control (zero-hour) larvae were washed and then collected for subsequent RNA extraction. For longer starvation, the sucrose media was changed daily. For complete starvation, larvae were transferred to paper moistened with Ringers solution. For amino acid starvation, larvae were transferred to fly media lacking protein or amino acids. Overexpression of PI3K and Myc transgenes was performed using the heat-shock flp-out method [42]. Transgenes were induced by transferring larvae to a 37C room for 1.5 hrs and then returning flies to 25C for the indicated post-induction recovery times. In each case controls were similarly heat-shocked larvae lacking the UAS transgene. Our microarray analysis indicated that this protocol led to a ~20-30 fold induction of Myc mRNA and PI3K mRNA (Additional File 1). For analysis of TOR mutants, hatched larvae were transferred and raised in regular fly food for 48 h before being collected for RNA extraction. Control larvae were age-matched w^1118 ^larvae.

### RNA extraction and microarray analysis

For all microarray experiments, larvae were collected, snap-frozen and maintained at -80C prior to RNA extraction. Total RNA was extracted with TRIzol reagent (Invitrogen) followed by RNeasy (Qiagen) clear up. cRNA targets were generated and coupled to either Cy3 or Cy5 fluorophores according to 'FHCRC Genomics Resource DNA Array Laboratory' protocols. Expression profiles were performed using spotted microarrays constructed from release 1 (6 K, Figs 1, 2, 3A, B, and 4) or 1 and 2 (12 K, Fig 3C, D) of the *D. melanogaster *Gene Collection (GEO GPL4285 = '6 k' and GEO GPL1908 = '12 k'). Target label preparation and hybridization protocols are described elsewhere http://www.fhcrc.org/science/shared_resources/genomics/index.html. Hybridization and scanning were performed by the Fred Hutchinson Cancer Research Center Genomics Resource DNA Array Laboratory. Microarray images were quantified using GenePix Pro software (Axon Instruments).

### Statistics and Bioinformatics

Data were generated from four or five independent replicates, with at least 2 replicates for each dye orientation. Spot intensities were filtered and removed if the values did not exceed 250 units above background or if a spot was flagged as questionable by the GenePix Pro software (Molecular Devices Corporation, Sunnyvale, CA). Spot-level ratios were log2 transformed and normalized using centralization method using Microsoft excel program to correct for observed intra-array intensity-dependent ratio biasing. Significance analysis of microarrays (SAM, http://www-stat.stanford.edu/~tibs/SAM/) was used to select statistically significant data and a two-class paired test was conducted. A fold change threshold of 2-fold for starvation, PI3 kinase overexpression, TOR, and Myc mutants, and 1.7 fold-threshold for Myc overexpression, plus a false discovery rate (FDR) of <5% were set as cut off to identify genes with statistically significant changes in expression. Heat maps were made using Cluster and TreeView program. http://rana.lbl.gov/EisenSoftware.htm.

Clusters of genes with similar expression patterns were generated using Genesis program http://genome.tugraz.at. Regulatory motifs were identified using MEME http://meme.sdsc.edu/meme4_1/cgi-bin/meme.cgi[43]. Upstream sequences of 500 bp plus 5'UTR of each gene from each cluster were used for motif search. GOMO (Gene Ontology for Motifs) [44] was used to analyze GO-terms significantly associated with genes containing motif1.

### GFP-reporter assays and primers

The upstream regions of selected genes (cluster A - CG768, CG1135, CG6311, CG32486; cluster B - CG8470, CG6459, CG9539) were amplified by PCR from genomic DNA isolated from wildtype flies and subcloned into Stinger GFP transformation vector (obtained from Drosophila Genome Resource Center). The 5× motif1 reporter genes were constructed by cloning five tandem repeats of Motif1 into a Stinger GFP transformation vector [45]. GFP mRNA levels were measured by quantitative real-time RT-PCR using RNA extracted from whole larvae.

## Authors' contributions

BAE and LL conceived of the project. BAE, LL and SSG designed the experiments. LL performed the microarray analyses and helped edit the manuscript. SSG performed the reporter assays in Fig 5, and drafted the manuscript. LL and SSG analyzed the data. All authors read and approved the final manuscript.

## Supplementary Material

Additional file 1 **List of genes affected in each microarray experiment**. Gene names and associated GO terms are shown. Click here for file

Additional file 2 **AA starvation induces a dynamic change in gene expression**. All genes altered by AA starvation were clustered into one of 16 different groups based on their expression profiles. The plots represent fold change in expression level at different starvation/reefed time-points for each gene in the cluster. The pink line represents the average expression profile for each cluster. Green shade indicates different starvation time-points, red shade indicates re-feed timepoints. Click here for file

Additional file 3 **Examples of gene classes affected by AA starvation in Drosophila larvae**. Heat maps depicting AA-starvation regulated genes involved lipid metabolism, carbohydrate metabolism, and TCA genes. Columns indicate expression changes at different AA starvation/re-feeding timepoints. Click here for file

Additional file 4 **Examples of gene classes affected by AA starvation in Drosophila larvae**. Heat maps depicting AA-starvation regulated genes in proteolysis or autophagy. Columns indicate expression changes at different AA starvation/re-feeding timepoints. Click here for file

Additional file 5 **Examples of gene classes affected by AA starvation in Drosophila larvae**. Heat maps depicting AA-starvation regulated genes involved in ribosome or protein synthesis. Columns indicate expression changes at different AA starvation/re-feeding timepoints. Click here for file

Additional file 6 **AA starvation has little effect on cell cycle genes**. Graphs depicting fold changes in AA starvation-induced genes, AA starvation-repressed genes and cell cycle genes, in response to AA starvation and subsequent re-feeding. AA starvation has little effect in the expression of cell cycle genes. Click here for file

Additional file 7 **Comparison of different starvation protocols on gene expression in Drosophila larvae**. Pie charts depicting overlap in both up-regulated (left) and down-regulated (right) gene expression following 24 h of a sucrose-only diet, complete starvation, or normal diet minus protein. Click here for file

Additional file 8 GO-enrichment for genes affected by PI3 kinase overexpression. Click here for file

Additional file 9 **GOMO analysis of GO terms associated with motif 1 containing genes**. The E-values and P-values are indicated for each GO term. Click here for file
