# Supplementary figures and images for: Nutritional control of gene expression in Drosophila larvae via TOR, Myc and a novel cis-regulatory element
Source: BMC Cell Biol. 2010 Jan 20;11:7. doi: 10.1186/1471-2121-11-7 (PMC2827378; doi:10.1186/1471-2121-11-7)

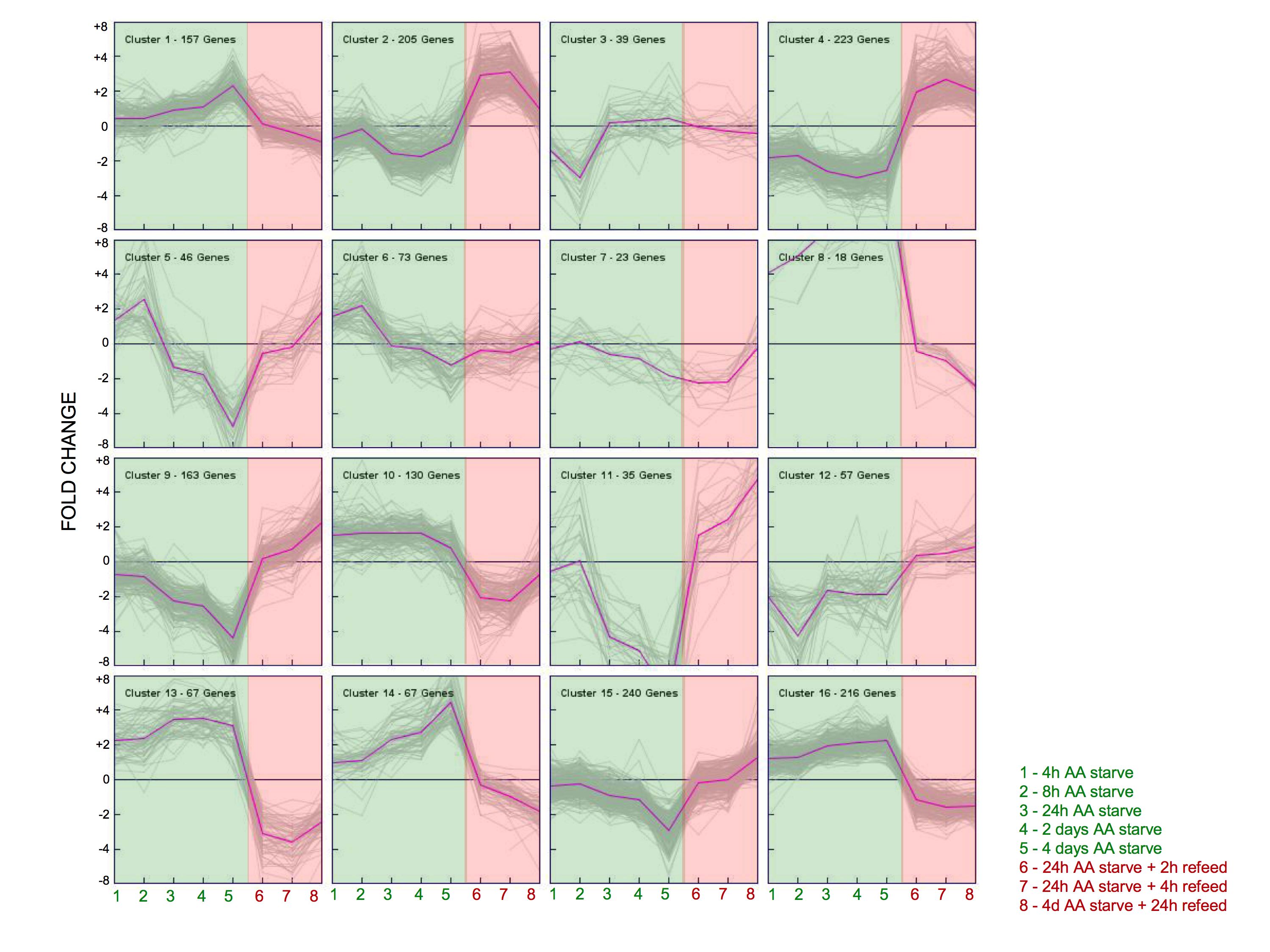

Supplement: Additional file 2 — AA starvation induces a dynamic change in gene expression. All genes altered by AA starvation were clustered into one of 16 different groups based on their expression profiles. The plots represent fold change in expression level at different starvation/reefed time-points for each gene in the cluster. The pink line represents the average expression profile for each cluster. Green shade indicates different starvation time-points, red shade indicates re-feed timepoints. [file 1471-2121-11-7-S2.JPEG]

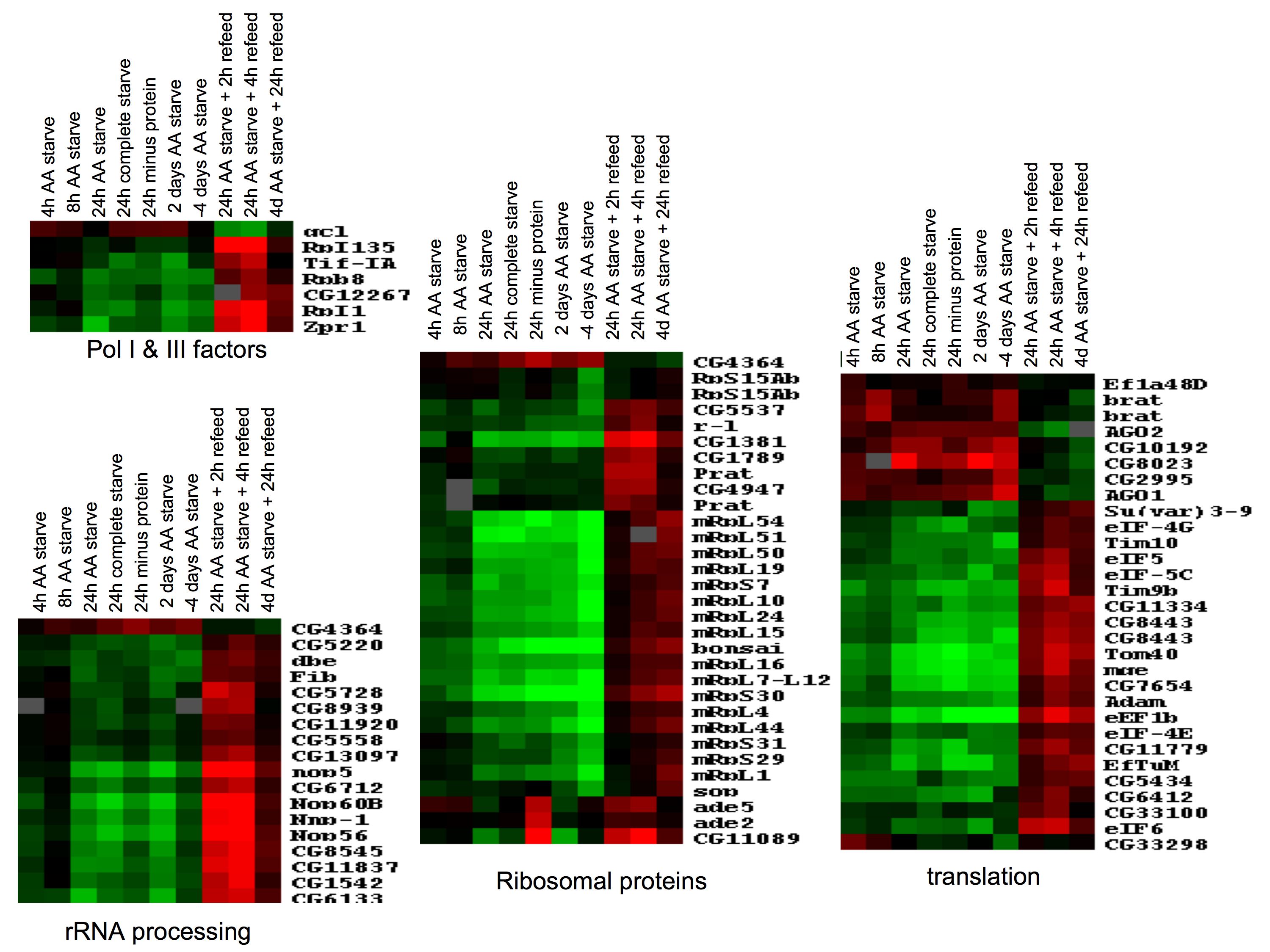

Supplement: Additional file 3 — Examples of gene classes affected by AA starvation in Drosophila larvae. Heat maps depicting AA-starvation regulated genes involved lipid metabolism, carbohydrate metabolism, and TCA genes. Columns indicate expression changes at different AA starvation/re-feeding timepoints. [file 1471-2121-11-7-S3.JPEG]

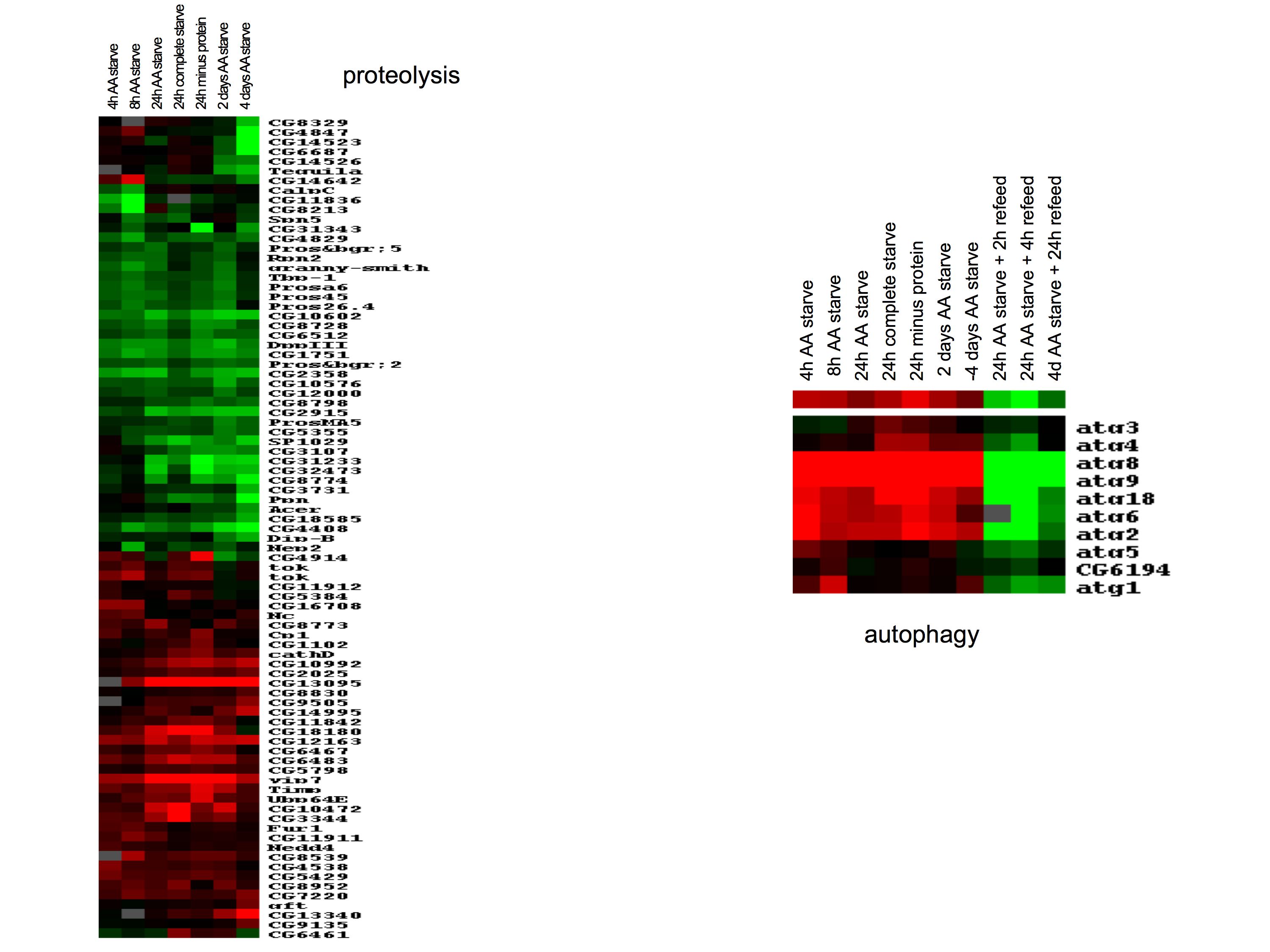

Supplement: Additional file 4 — Examples of gene classes affected by AA starvation in Drosophila larvae. Heat maps depicting AA-starvation regulated genes in proteolysis or autophagy. Columns indicate expression changes at different AA starvation/re-feeding timepoints. [file 1471-2121-11-7-S4.JPEG]

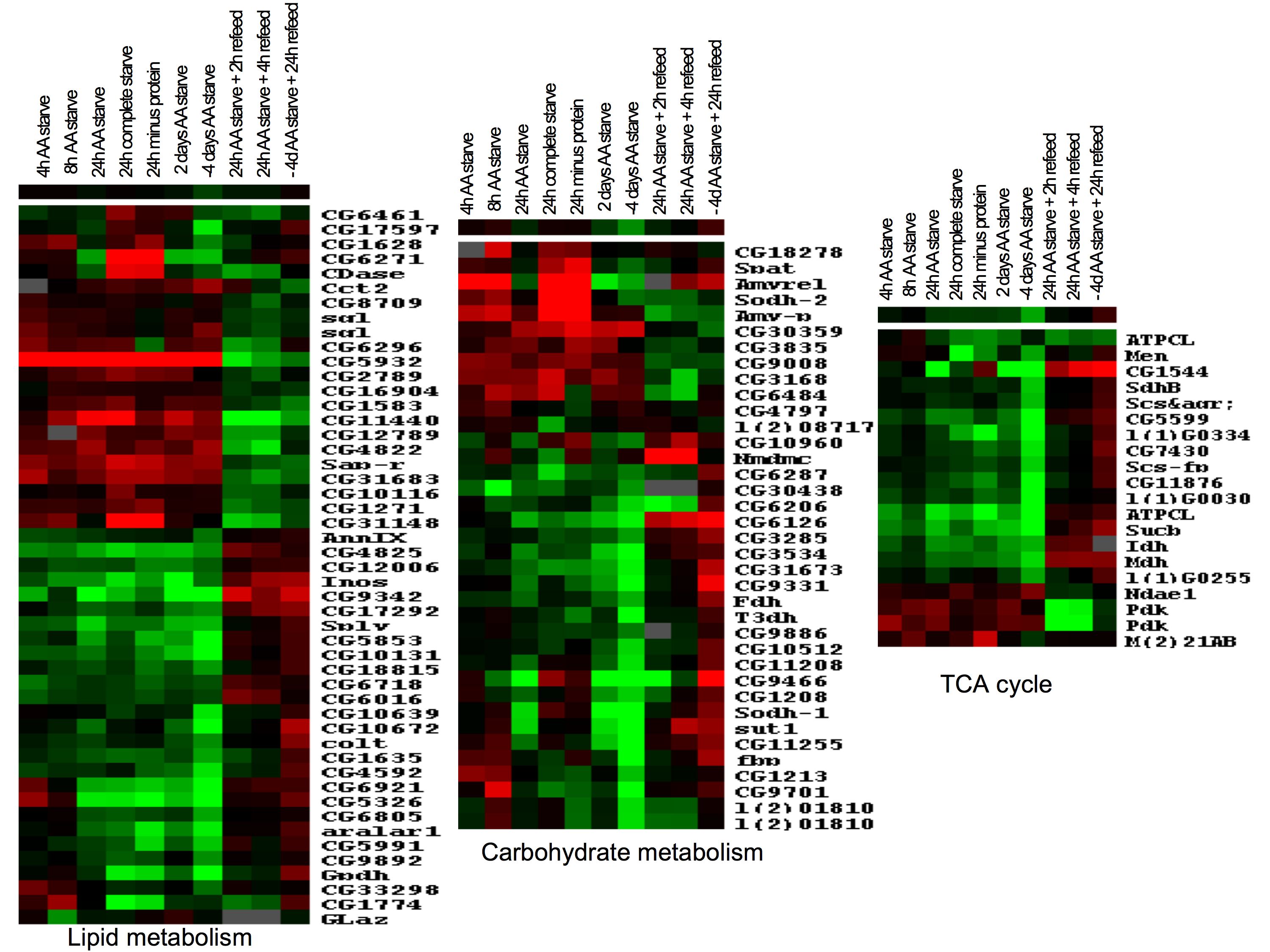

Supplement: Additional file 5 — Examples of gene classes affected by AA starvation in Drosophila larvae. Heat maps depicting AA-starvation regulated genes involved in ribosome or protein synthesis. Columns indicate expression changes at different AA starvation/re-feeding timepoints. [file 1471-2121-11-7-S5.JPEG]

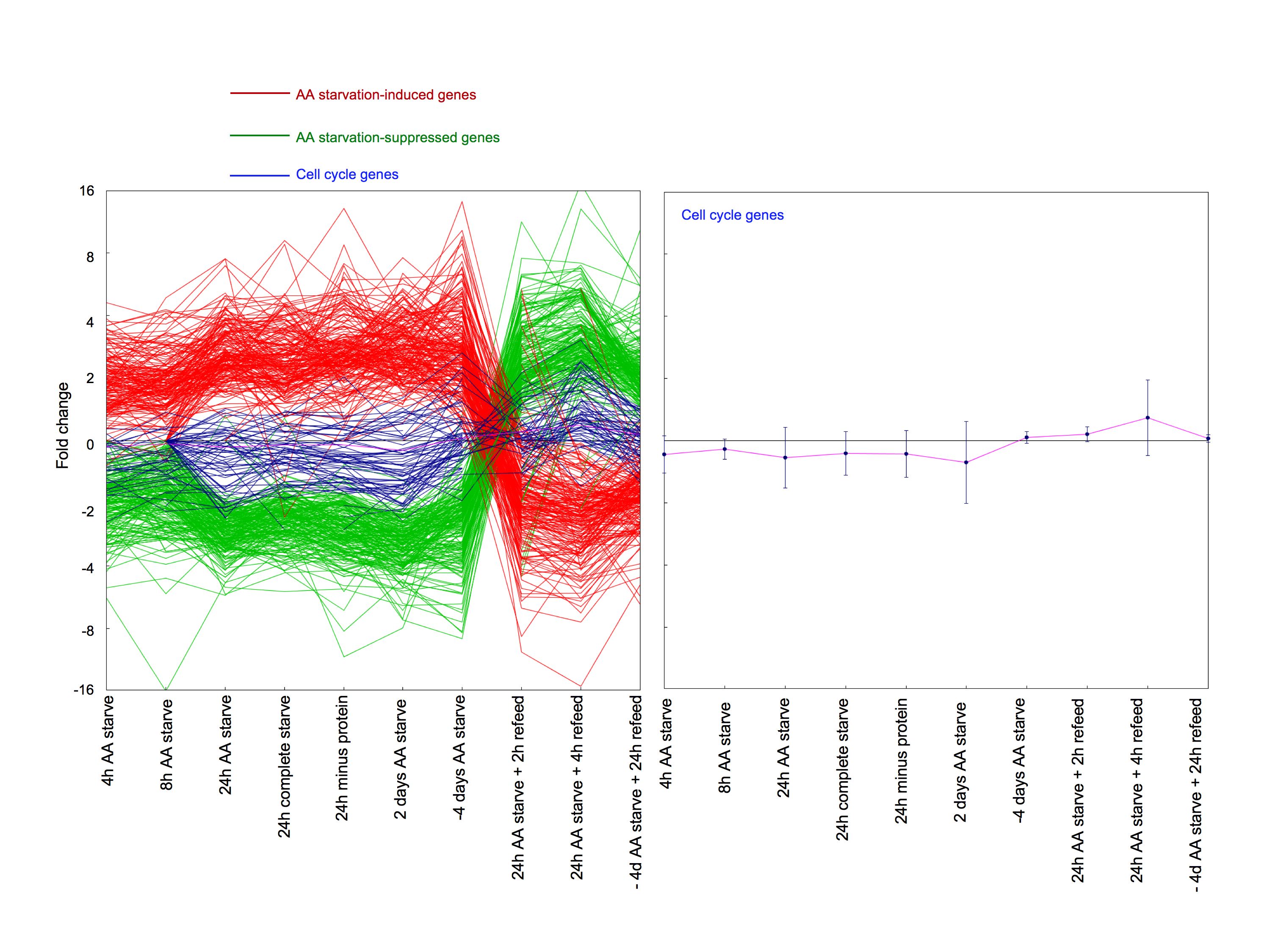

Supplement: Additional file 6 — AA starvation has little effect on cell cycle genes. Graphs depicting fold changes in AA starvation-induced genes, AA starvation-repressed genes and cell cycle genes, in response to AA starvation and subsequent re-feeding. AA starvation has little effect in the expression of cell cycle genes. [file 1471-2121-11-7-S6.JPEG]

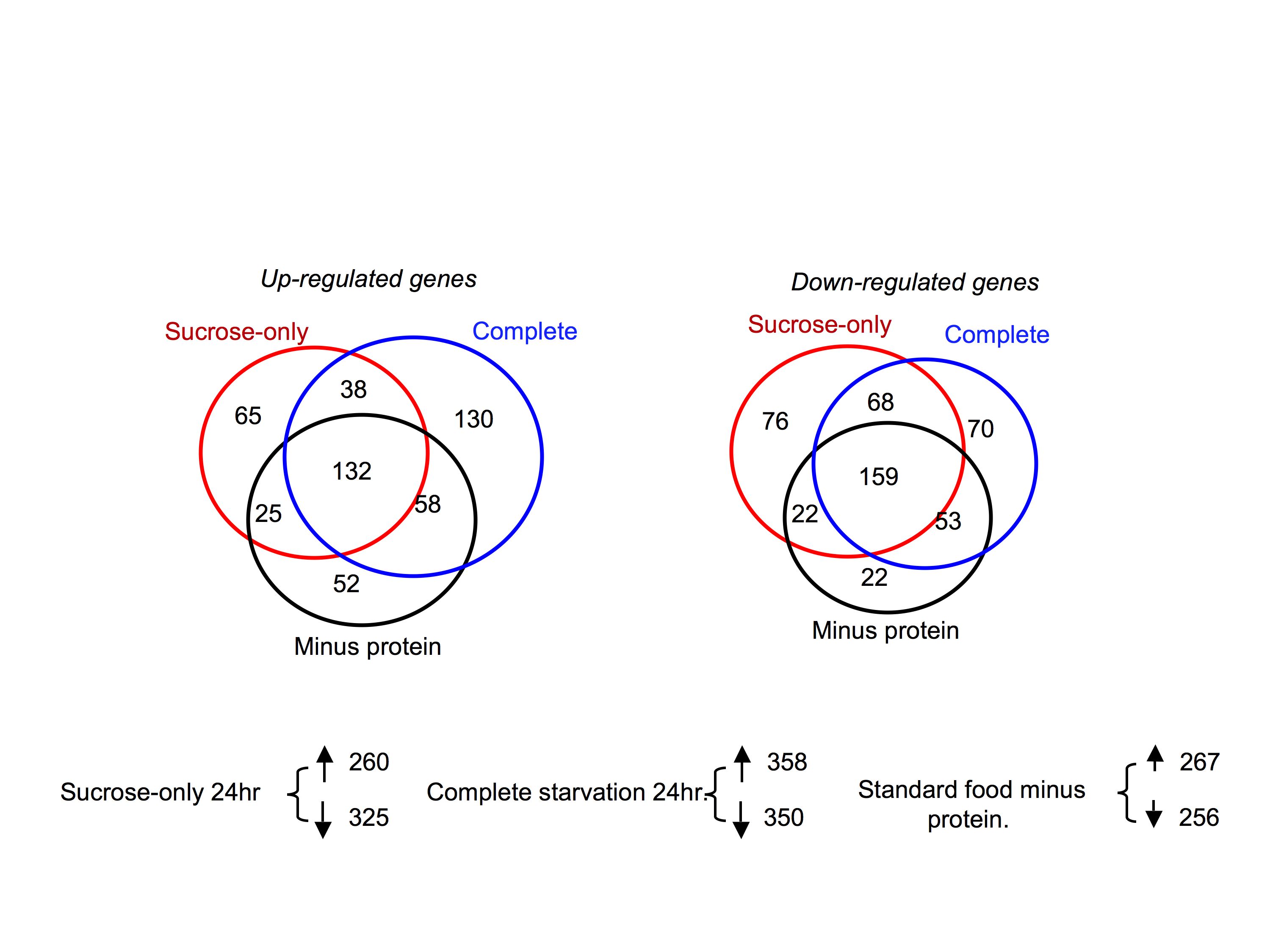

Supplement: Additional file 7 — Comparison of different starvation protocols on gene expression in Drosophila larvae. Pie charts depicting overlap in both up-regulated (left) and down-regulated (right) gene expression following 24 h of a sucrose-only diet, complete starvation, or normal diet minus protein. [file 1471-2121-11-7-S7.JPEG]
