# Supplementary material for: Nutritional control of gene expression in Drosophila larvae via TOR, Myc and a novel cis-regulatory element
Source: BMC Cell Biol. 2010 Jan 20;11:7. doi: 10.1186/1471-2121-11-7 (PMC2827378; doi:10.1186/1471-2121-11-7)
Supplement: Additional file 8 — GO-enrichment for genes affected by PI3 kinase overexpression. [file 1471-2121-11-7-S8.PDF]

Additional File 8: GO-term enrichment for genes affected by PI3K overexpression

**Biological process (GO terms – level 4 category)**	**Percentage of upregulated genes**	**Percentage of downregulated genes**
GO:0044260 carbohydrate metabolic process	42.44	32.47
GO:0019538 protein metabolic process	37.21	29.87
GO:0006091 generation of precursor metabolites and energy	26.74	14.29
GO:0044249 cellular biosynthetic process	19.19	12.99
GO:0043283 biopolymer metabolic process	18.6	22.08
GO:0009308 amine metabolic process	16.86	7.79
GO:0006139 nucleobase, nucleoside, nucleotide and nucleic acid metabolic process	16.28	12.99
GO:0006082 organic acid metabolic process	13.95	6.49
GO:0005975 carbohydrate metabolic process	12.79	7.79
GO:0006810 transport	11.63	10.39
GO:0006519 amino acid and derivative metabolic process	11.63	5.19
GO:0044248 cellular catabolic process	6.4	5.19
GO:0006629 lipid metabolic process	5.23	6.49
