# Supplementary material for: Nutritional control of gene expression in Drosophila larvae via TOR, Myc and a novel cis-regulatory element
Source: BMC Cell Biol. 2010 Jan 20;11:7. doi: 10.1186/1471-2121-11-7 (PMC2827378; doi:10.1186/1471-2121-11-7)
Supplement: Additional file 9 — GOMO analysis of GO terms associated with motif 1 containing genes. The E-values and P-values are indicated for each GO term. [file 1471-2121-11-7-S9.PDF]

Go term	E-value	q-value	GO definition
GO:0022402	1.77E-06	2.40E-07	biological_process: cell cycle process
GO:0006139	4.76E-06	4.83E-07	biological_process: nucleobase, nucleoside, nucleotide and nucleic acid metabolic process
GO:0007049	6.04E-06	4.95E-07	biological_process: cell cycle
GO:0000278	6.95E-06	5.13E-07	biological_process: mitotic cell cycle
GO:0022403	1.71E-05	6.93E-07	biological_process: cell cycle phase
GO:0007010	1.90E-05	7.34E-07	biological_process: cytoskeleton organization and biogenesis
GO:0007017	3.77E-05	1.27E-06	biological_process: microtubule-based process
GO:0000226	6.25E-05	1.88E-06	biological_process: microtubule cytoskeleton organization and biogenesis
GO:0000279	6.95E-05	2.01E-06	biological_process: M phase
GO:0007051	2.42E-04	6.78E-06	biological_process: spindle organization and biogenesis
GO:0007052	3.89E-04	1.05E-05	biological_process: mitotic spindle organization and biogenesis
GO:0009987	1.35E-03	3.32E-05	biological_process: cellular process
GO:0043170	1.50E-03	3.46E-05	biological_process: macromolecule metabolic process
GO:0008104	3.86E-03	7.83E-05	biological_process: protein localization
GO:0044237	4.07E-03	7.92E-05	biological_process: cellular metabolic process
GO:0051641	8.34E-03	1.50E-04	biological_process: cellular localization
GO:0043283	8.58E-03	1.51E-04	biological_process: biopolymer metabolic process
GO:0044238	8.83E-03	1.52E-04	biological_process: primary metabolic process
GO:0033036	1.15E-02	1.87E-04	biological_process: macromolecule localization
GO:0016070	1.29E-02	2.06E-04	biological_process: RNA metabolic process
GO:0016043	1.68E-02	2.57E-04	biological_process: cellular component organization and biogenesis
GO:0051649	3.68E-02	5.33E-04	biological_process: establishment of localization in cell
GO:0006396	4.53E-02	6.45E-04	biological_process: RNA processing
GO:0006260	5.90E-02	8.12E-04	biological_process: DNA replication
GO:0006996	6.02E-02	8.15E-04	biological_process: organelle organization and biogenesis
GO:0006259	1.24E-01	1.64E-03	biological_process: DNA metabolic process
GO:0006277	1.38E-01	1.78E-03	biological_process: DNA amplification
GO:0008152	2.05E-01	2.60E-03	biological_process: metabolic process
GO:0043285	2.94E-01	3.67E-03	biological_process: biopolymer catabolic process
GO:0016568	5.66E-01	6.20E-03	biological_process: chromatin modification
GO:0006414	5.73E-01	6.20E-03	biological_process: translational elongation
GO:0007307	6.38E-01	6.81E-03	biological_process: eggshell chorion gene amplification
GO:0008283	7.89E-01	8.18E-03	biological_process: cell proliferation
GO:0009057	7.84E-01	8.18E-03	biological_process: macromolecule catabolic process
GO:0065008	7.96E-01	8.18E-03	biological_process: regulation of biological quality
GO:0051726	8.69E-01	8.50E-03	biological_process: regulation of cell cycle
GO:0016192	8.86E-01	8.56E-03	biological_process: vesicle-mediated transport
GO:0007306	9.39E-01	8.97E-03	biological_process: eggshell chorion formation
GO:0005622	4.93E-08	2.26E-08	cellular_component: intracellular
GO:0043234	5.58E-08	2.26E-08	cellular_component: protein complex
GO:0044446	2.64E-07	5.40E-08	cellular_component: intracellular organelle part
GO:0044422	2.66E-07	5.40E-08	cellular_component: organelle part
GO:0044424	6.47E-07	1.05E-07	cellular_component: intracellular part
GO:0043229	8.99E-06	5.21E-07	cellular_component: intracellular organelle
GO:0043226	8.75E-06	5.21E-07	cellular_component: organelle
GO:0043231	1.39E-05	6.58E-07	cellular_component: intracellular membrane-bounded organelle
GO:0032991	1.31E-05	6.58E-07	cellular_component: macromolecular complex
GO:0043227	1.60E-05	6.82E-07	cellular_component: membrane-bounded organelle
GO:0044428	5.05E-05	1.58E-06	cellular_component: nuclear part
GO:0044445	1.52E-03	3.46E-05	cellular_component: cytosolic part
GO:0005737	1.96E-03	4.19E-05	cellular_component: cytoplasm
GO:0022626	4.10E-03	7.92E-05	cellular_component: cytosolic ribosome
GO:0005634	4.40E-03	8.30E-05	cellular_component: nucleus
GO:0031090	9.11E-03	1.54E-04	cellular_component: organelle membrane
GO:0031981	4.97E-02	6.95E-04	cellular_component: nuclear lumen
GO:0005623	3.23E-01	3.91E-03	cellular_component: cell
GO:0044464	3.23E-01	3.91E-03	cellular_component: cell part
GO:0043232	3.81E-01	4.48E-03	cellular_component: intracellular non-membrane-bounded organelle
GO:0043228	3.81E-01	4.48E-03	cellular_component: non-membrane-bounded organelle
GO:0030529	4.81E-01	5.58E-03	cellular_component: ribonucleoprotein complex
GO:0044444	5.04E-01	5.76E-03	cellular_component: cytoplasmic part
GO:0031974	5.51E-01	6.12E-03	cellular_component: membrane-enclosed lumen
GO:0043233	5.51E-01	6.12E-03	cellular_component: organelle lumen
GO:0022625	8.53E-01	8.47E-03	cellular_component: cytosolic large ribosomal subunit
GO:0033279	8.55E-01	8.47E-03	cellular_component: ribosomal subunit
GO:0005840	1.00E+00	9.43E-03	cellular_component: ribosome
GO:0017111	3.41E-06	3.95E-07	molecular_function: nucleoside-triphosphatase activity
GO:0016462	6.10E-06	4.95E-07	molecular_function: pyrophosphatase activity
GO:0000166	7.97E-06	5.21E-07	molecular_function: nucleotide binding
GO:0032555	1.46E-05	6.58E-07	molecular_function: purine ribonucleotide binding
GO:0032553	1.46E-05	6.58E-07	molecular_function: ribonucleotide binding
GO:0016817	3.49E-05	1.23E-06	molecular_function: hydrolase activity, acting on acid anhydrides
GO:0016818	3.49E-05	1.23E-06	molecular_function: hydrolase activity, acting on acid anhydrides, in phosphorus-containing anhydrides
GO:0017076	4.84E-05	1.57E-06	molecular_function: purine nucleotide binding
GO:0003824	6.78E-04	1.78E-05	molecular_function: catalytic activity
GO:0004386	1.34E-03	3.32E-05	molecular_function: helicase activity
GO:0005524	1.54E-03	3.46E-05	molecular_function: ATP binding
GO:0032559	1.89E-03	4.15E-05	molecular_function: adenyl ribonucleotide binding
GO:0030554	3.31E-03	6.90E-05	molecular_function: adenyl nucleotide binding
GO:0016887	4.76E-03	8.79E-05	molecular_function: ATPase activity
GO:0042623	1.11E-02	1.83E-04	molecular_function: ATPase activity, coupled
GO:0005515	1.33E-02	2.08E-04	molecular_function: protein binding
GO:0016787	1.85E-02	2.78E-04	molecular_function: hydrolase activity
GO:0008026	3.19E-02	4.70E-04	molecular_function: ATP-dependent helicase activity
GO:0003713	1.32E-01	1.73E-03	molecular_function: transcription coactivator activity
GO:0003724	8.49E-01	8.47E-03	molecular_function: RNA helicase activity

Additional File 9. GOMO analysis of GO-terms associated with Motif 1-containing genes.
